# SARS-CoV-2 Positive and Isolated at Home: Stress and Coping Depending on Psychological Burden

**DOI:** 10.3389/fpsyt.2021.748244

**Published:** 2021-11-22

**Authors:** Elias Kowalski, Axel Schneider, Stephan Zipfel, Andreas Stengel, Johanna Graf

**Affiliations:** ^1^Department of Psychosomatic Medicine and Psychotherapy, University Hospital Tübingen, Tübingen, Germany; ^2^Health Department Freudenstadt, Freudenstadt, Germany; ^3^Charité Center for Internal Medicine and Dermatology, Department for Psychosomatic Medicine, Charité-Universitätsmedizin Berlin, Corporate Member of Freie Universität Berlin, Humboldt-Universität zu Berlin and Berlin Institute of Health, Berlin, Germany

**Keywords:** cross-sectional study, COVID-19, mental health, patient, psychological, quarantine

## Abstract

**Objective:** The SARS-CoV-2 pandemic has led to pronounced health changes, especially for those infected and psychologically burdened. This cross-sectional study examined the stress experience and coping strategies during home isolation of SARS-CoV-2 infected individuals and analyzed differences regarding psychological burden.

**Methods:** SARS-CoV-2 infected respondents were recruited by telephone and completed an online survey during their home isolation. This questionnaire assessed sociodemographic aspects, somatic factors, psychological burden (depressive symptoms, anxiety, and somatic symptom disorder), perceived stress and coping behavior during the home isolation.

**Results:** Out of 838 SARS-CoV-2 infected individuals during the study period, 648 were contacted and 224 home-isolated respondents were included in the study. Disgrace, social restrictions, job fear, health concerns, and infectiousness could be explored as stressors during the home isolation. Fifty-four percent experienced psychological burden. SARS-CoV-2 infected and home-isolated individuals with psychological burden perceived significant stressors more strongly (*p* < 0.001, *r* = 0.5) and coped significantly less (*p* < 0.001, *r* = 0.3) with their infection and home isolation compared to SARS-CoV-2 infected individuals without psychological burden.

**Conclusion:** SARS-CoV-2 infected individuals with psychological burden experienced higher stressors and were unable to cope adaptively with home isolation. Therefore, a general and standardized screening procedure for psychological burden should be established. SARS-CoV-2 infected individuals with psychological burden should receive targeted support with professional help in the areas of stress experience and coping skills during their home isolation and beyond to avoid long-term consequences.

## Introduction

Since the World Health Organization (WHO) declared the impact of the SARS-CoV-2 virus on humanity a pandemic of international scope on March 11th 2020 (1), major health-related changes have occurred worldwide. Media reports and policy decisions have concentrated on the daily case numbers of SARS-CoV-2-confirmed cases (over 230 million) and SARS-CoV-2 deceased (over 4.8 million) (2). SARS-CoV-2 infection is now a common disease. In a German study on patients (*n* = 301,290) with SARS-CoV-2 associated symptoms in general practices, the prevalence of SARS-CoV-2 infection was 13.8% (3).

The infected are those who, on the one hand, experience the health threat most acutely and, on the other hand, are most urgently exposed to the harshest countermeasures in the fight against the pandemic. The key element of global pandemic response is to break chains of infection (“flatten the curve”). In Germany, the public health institute (Robert Koch Institute) develops the national guidelines for the work of the individual health authorities. Infected persons must be isolated for at least 14 days from the onset of symptoms or from the date of testing positive. Isolation is the separation of confirmed infected persons. This definition differs from the concept of quarantine, which is the separation of contact persons who are suspected of being infected because they had contact with confirmed infected persons. The situations between quarantine and isolation differ significantly (4). For example, infected persons are exposed to different stressors (such as the great concern of having infected others) than their contacts, who are confronted with the uncertainty of whether they will still become ill. However, infected individuals and their contacts are not allowed to leave their homes during separation except for SARS-CoV-2 swabs and in emergencies. It is also recommended that they separate themselves from their own household members. In Germany, this containment strategy is coordinated by the local district health offices.

Meanwhile, many studies have dealt with the psychological burden due to SARS-CoV-2 infection. In a meta-analysis of 22 studies (*n* = 4,318), 38% of SARS-CoV-2 infected persons had depression and an equal number had anxiety (by reaching an appropriate cut-off value of screening questionnaires) (5). Pre-existing mental illness is associated with increased psychological burden due to SARS-CoV-2 infection (6). A cross-sectional study on hospitalized patients (*n* = 281) showed that patients with psychiatric disorder were vulnerable to the development of anxiety symptoms during their SARS-CoV-2 infection (6). Further, a small study (*n* = 30) on hospitalized SARS-CoV-2 infected patients suggested that increased anxiety and depression are associated with worse, or even more lethal outcomes (7).

Not only the SARS-CoV-2 infection itself, but also the isolation procedure triggers psychological burden. A current study from Bangladesh (*n* = 5,792) found a very high prevalence of depression (85.9%) among SARS-CoV-2 infected persons in isolation (8). Since now, the focus of the SARS-CoV-2 isolation studies has been on prevalence assessment of psychological burden among persons with a SARS-CoV-2 infection. However, to adequately support infected people in isolation, a deep understanding of the stressors and coping mechanism during the isolation is very important and still missing. Only few studies have evaluated stressors during separation measures of previous epidemics. They showed stressors like boredom and lack of self-control (9–13). There is only one study (*n* = 64) that has assessed the stressors of separation measures through SARS-CoV-2 isolation or quarantine in the setting at home (14). Qualitative interviews explored various stigmatization processes that led to impaired quality of life. Coping as the ability to manage stressful situations is a very important skill to overcome challenging situations as pandemics. Only three studies (*n* = 66, 84, and 100) have addressed the coping strategies of SARS-CoV-2 infected and hospitalized patients (15–17). The patients coped for example with positive reframing, emotional support, and communication with their families (15–17).

Essential points for understanding the isolation of SARS-CoV-2 infected persons are still unexplored. This particularly concerns the isolation at home. Most studies that have addressed stress and coping in SARS-CoV-2 isolation have looked at the situation of hospitalized patients (15–17). However, most SARS-CoV-2 infected persons spend their isolation at home. At home, the overall situation is very different compared to the hospitalized isolation. There is no around-the-clock medical care at home, no predefined daily structure, no symptom management etc. that might support adaptive coping in infected patients. Instead, SARS-CoV-2 infected individuals in isolation live in constant fear of infecting their household members due to inadequate isolation options and are left to cope with isolation on their own. Furthermore, isolation of infected individuals differs from quarantine of contact persons in many ways. The previous study on isolation at home did not consider this separately (14), although stress experience and coping strategies certainly differ significantly between isolation and quarantine. In summary, there is no data yet that examines the impact of isolation at home on mental health in people infected with SARS-CoV-2. However, this is important to develop care concepts with the aim to enhance coping strategies despite their home isolation and managing a SARS-CoV-2 infection. This is the first cross-sectional study of SARS-CoV-2 infected individuals that evaluates [1] the experienced psychological burden and stressors in home isolation, [2] the coping strategies during home isolation, and [3] the differences in stress experience and coping behavior regarding psychological burden. We tested the hypothesis that psychological burden is high during home isolation and that psychologically burdened individuals experience increased stress associated with lower coping strategies.

## Materials and Methods

### Study Design and Recruitment

The study population consists of SARS-CoV-2 infected and home isolated persons within the jurisdiction of the Freudenstadt Health Department (Germany). All infected persons had at least one positive SARS-CoV-2 PCR result at the time of the survey. The survey was conducted between January 29th and April 12th 2021. During this period, all infected persons whose positive PCR result was received by the Freudenstadt Health Department by March 31th 2021 at the latest were contacted by telephone during their first days of home isolation (*n* = 838).

During the phone call, patients were screened if they were cognitively able to participate in the study. The screening procedure was conducted with general questions regarding to the situation and person. If people expressed interest in the anonymous investigation, an email was sent directly after the phone call (*n* = 478) with further information about the study and with an invitation link to the online survey (EFS Survey) (18).

**Figure 1:**
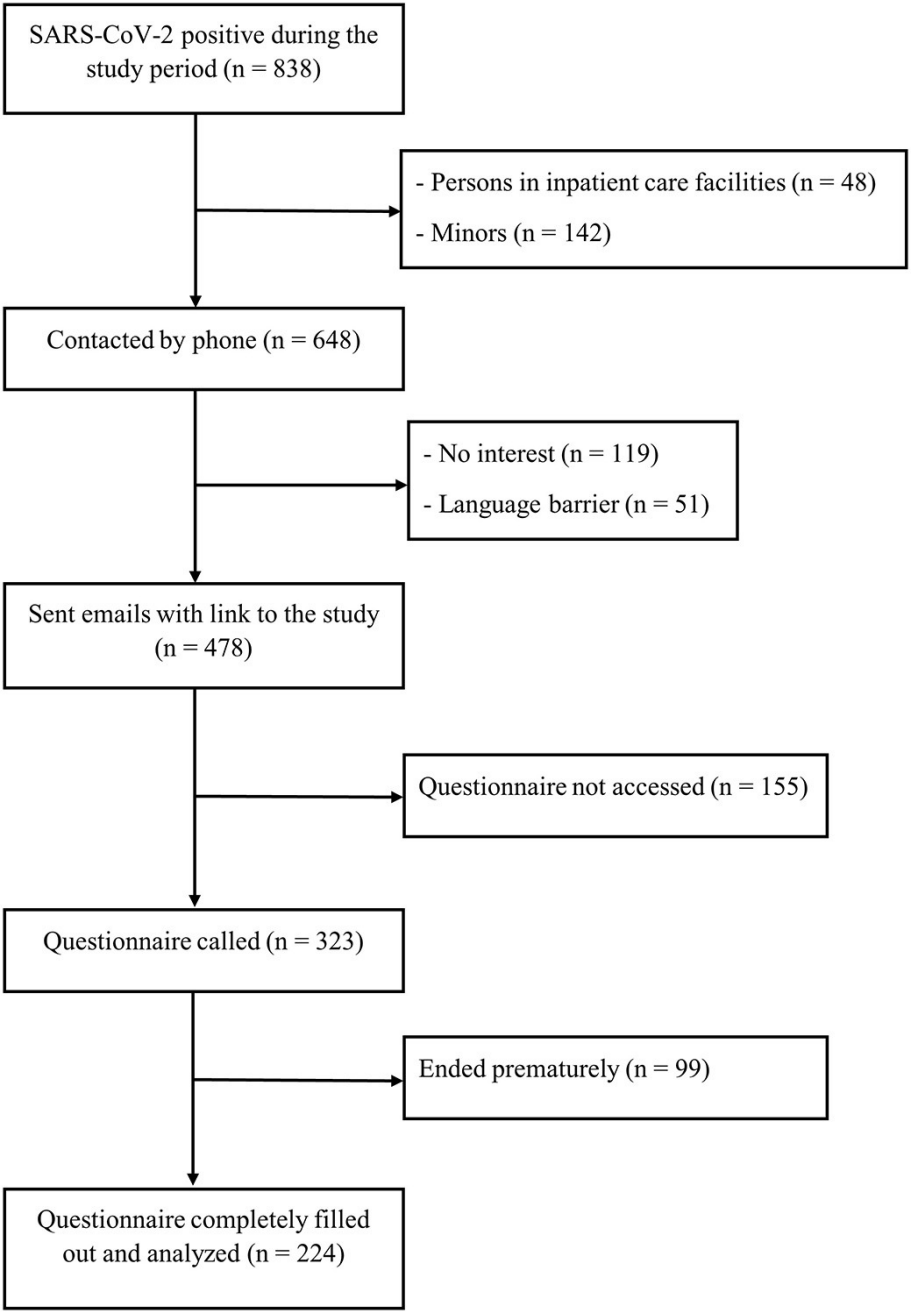
Flow chart of study recruitment.

Patients completed the survey while still being in their home isolation. To prevent multiple completion, repeated access *via* the same IP address was blocked. All participants who did not finish the questionnaire were excluded (*n* = 99, from which 80.8% quit at the very beginning). Two hundred and twenty-four SARS-CoV-2 infected and home isolated respondents could be included in the study (see Figure 1). Upon completion of the online survey, all participants received information for further psychological support during the isolation at home.

### Procedures/Ethics Statement

At the beginning of the online survey, consent forms were obtained for data protection, data processing and data analysis. Study participation was voluntary and anonymous, took about 40 min, and the questionnaire could be discontinued at any time without negative consequences for the participants. The local ethics committee approved the study (073/2021BO2).

### Measures

#### Demographics, Somatic Parameters, and Subjective Support During Home Isolation

Age, gender, marital status, number of children, number of household members, size of residence, nationality, and highest level of education were assessed. Self-reported SARS-CoV-2 associated symptoms, risk factors for a more severe SARS-CoV-2 course according to the Robert Koch Institute (German public health institute) and the subjective assessment of the current and worst physical condition (0 = not ill at all to 100 = very severely ill) were assessed. Four self-generated items were used to assess subjective support (“*How much do you feel supported by your social environment/employer/family doctor/health office during your isolation?*”; 0 = no support at all to 100 = full support). Total support score was formed which has an acceptable reliability in the current sample (Cronbach's α = 0.684).

#### Patient Health Questionnaire

The Patient Health Questionnaire 8 (PHQ-8) originates from the Patient Health Questionnaire (19) and captures the module of depressiveness (20). Depressive symptomatology and depression severity are measured by a retrospective self-assessment of the past 2 weeks using a 4-point Likert scale (0 = not at all to 3 = nearly every day) (21). Clinically significant depressive symptoms can be assumed from a sum score of 10 (range = 0–24) (21). The PHQ-8 showed very good reliability in the current sample (Cronbach's α = 0.858).

#### Generalized Anxiety Disorder Scale

The Generalized Anxiety Disorder Scale 7 (GAD-7) is also taken from the Patient Health Questionnaire (19) and contains seven items assessing generalized anxiety disorder and general anxiety in a valid and standardized way (22, 23). A 4-point Likert scale screens for frequency of anxiety in the past 2 weeks (24). A sum score of 10 or greater indicates at least moderate anxiety (range = 0–21) (24). The GAD-7 showed very good reliability in the current sample (Cronbach's α = 0.882).

#### Somatic Symptom Disorder-B Criteria Scale

The Somatic Symptom Disorder-B Criteria Scale questionnaire is used to detect somatic symptom disorders based on DSM in a valid way (25–31). The 12-item questionnaire is divided into cognitive, affective, and behavioral aspects (32). A 5-point Likert scale (0 = never to 5 = very often) is used to assess somatoform disorders. Depending on age and gender, the sum score (range = 0–48) is recategorized into at least medium (80% quantile) psychological distress using a quantile regression based on norm values (32). The SSD-12 exhibited very good reliability in the current sample (Cronbach's α = 0.927).

#### Perceived Stress Questionnaire

The Perceived Stress Questionnaire (PSQ-20) is a short form of the PSQ-30 and measures the subjective perception, evaluation, and processing of stressors over the last 4 weeks using a 4-point Likert scale (1 = almost never to 4 = most of the time) (33). The items are divided into four scales: worries, tension, joy, and demands (34). The four scales are recoded to a scale rank from 0 to 100 (low to high expression) and after inverting the joy category, a total score was formed. The total score showed very good reliability in the current sample (Cronbach's α = 0.903).

#### Stress and Coping Inventory

The Stress and Coping Inventory records stress symptoms and various coping strategies (35). In this study, only the independent coping modules positive thinking (e.g., “*I tell myself that stress and pressure also have their good sides*”), active coping (e.g., “*I do everything I can to prevent stress in the first place*”), and social support (e.g., “*When I get under pressure, I have people to help me*”) were recorded (35). The remaining 12 items were recorded using a 4-point Likert scale (1 = strongly disagree to 4 = strongly agree) (35, 36). The SCI showed good to very good reliability regarding all scales in the current sample (range of Cronbach's α: 0.717–0.827).

#### Specific Stressors Arising From Isolation and SARS-CoV-2 Infection

Sixteen items were created as home isolation- and SARS-CoV-2 infection-specific stressors based on the authors' extensive practical expertise witnessing the experiences of over 5,000 SARS-CoV-2 infected individuals. The items could be intuitively rated using a slider (0 = strongly disagree to 100 = strongly agree). A principal factor analysis with 16 items was performed with the help of an oblique rotation. Sampling adequacy was confirmed with Kaiser–Meyer–Olkin criterion (KMO = 0.773, range of KMO-values for individual items: 0.596–0.911) (37). According to Kaiser's criterion (eigenvalues > 1) (38, 39) and the scree plot, five factors were drawn, covering a total explained variance of 55.3%: disgrace, social restrictions, job fear, health concerns and infectiousness (e.g., “*I am worried that the disease will take a severe course in me*,” “*I feel guilty because I might have infected those around me*”). The absolute values of factor loadings were between 0.318 and 0.896 according to oblique rotation. All subscales had good to very good reliabilities in the current sample (range of Cronbach's α: 0.729–0.804). Total score and subscores were formed.

#### Coping of Isolated SARS-CoV-2 Infected Individuals

Twenty-four items were created by the expert team to assess how isolated SARS-CoV-2 infected people cope with this specific situation (e.g., “*with my isolation I do something for society*,” “*I maintain social contacts*”). The questions could be answered intuitively with a slider (0 = do not agree at all to 100 = agree completely). To create suitable subscales/constructs, various methods of exploratory factor analysis were applied. No constellation of factors could be found that appeared to be satisfactorily coherent both statistically and in terms of content. Consequently, a total score of 14 items was formed, which has a very good reliability in the current sample (Cronbach's α = 0.857). The discriminatory power of the individual items was good (range of corrected item-total correlation: 0.385–0.645).

### Statistical Analyses

Data were analyzed using SPSS (Version 27). Descriptive statistics, means, standard deviations, frequencies and percentages were examined. 20.1% respondents had at least one missing value. The Little's MCAR test (40) (*p* = 0.463) and logistic regression estimation (41) with binary coded missing variables as dependent variables (0.063 ≥ *p* < 1.000) showed that the missing data were subject to a missing completely at random mechanism. The Fully Conditional Specification Method of Multiple Imputation was used to impute the missing values (20 imputations were performed) (42). Normal distribution was tested, and the dataset was found to be not normally distributed, so the Mann–Whitney *U*-test was used for the significance tests (2-sided, *p* < 0.05). All respondents who had a somatic symptom disorder or/and depressive symptoms or/and anxiety were grouped together as the category psychological burden. Thus, psychological burden was defined as reaching at least one of the cut-off scores of PHQ-8, GAD-7, or SSD-12. In the significance tests, respondents with psychological burden were always compared to respondents without psychological burden.

## Results

### Sample Description

#### Sociodemographic

Of the 224 respondents, 52.7% were female and 43.3% were male. The mean age was 41 years (*SD* = 14.6). Further sociodemographic characteristics are listed in Table 1.

**Table 1:** Study population characteristics: sociodemographic (*n* = 224).

**Sociodemographic characteristics**	***N***	**%**
**Gender**
Female	118	52.7
Male	106	47.3
**Age (years)**
Mean	41.0	
SD	14.6	
Range	18–80	
**Marital status**
Single	72	32.1
Married/registered civil partnership	140	62.5
Divorced/dissolved civil partnership	11	4.9
Widowed	1	0.4
**Children**
No	79	35.3
Yes	145	64.7
**Number of total household members**
1	28	12.5
2	74	33.0
>2	122	54.5
**Residence size**
<500	23	10.3
500–1,000	39	17.4
1,000–5,000	92	41.1
5,000–10,000	35	15.6
>10,000	35	15.6
**Nationality**
German	196	87.5
Non-German	28	12.5
**Highest educational level**
Lower secondary education	18	8.0
Higher secondary education	127	56.7
University entrance qualification	23	10.3
University education	56	25.0

#### SARS-CoV-2 Associated Characteristics

On average, the survey was completed on the 12th day of isolation. One-hundred percent of the study participants spent their isolation at home (=private isolation). 93.3% of respondents had SARS-CoV-2 symptoms, with a mean of 6.1 symptoms (*SD* = 3.8, *Range* = 0–16). 45.1% had at least one SARS-CoV-2 associated risk factor. Additional SARS-CoV-2 associated characteristics can be found in Table 2.

**Table 2:** Study population characteristics: SARS-CoV-2-associated characteristics (*n* = 224).

**SARS-CoV-2-associated characteristics**	***N***	**%**
**Isolation days at the time of the survey**
Mean	12.7	
SD	4.6	
Range	0–29	
**Place of isolation**
Private	224	100.0
Other	5	2.2
**SARS-CoV-2 associated symptoms**
Yes	209	93.3
No	15	6.7
**Most frequent SARS-CoV-2 associated symptoms**
Headache	143	63.8
Sniffles	141	62.9
Cough	138	61.6
Increased sleepiness/tiredness	129	57.6
Pain in the limbs	108	48.2
Disturbance of the sense of smell	101	45.1
**SARS-CoV-2 associated risk factors**
Yes	101	45.1
No	123	54.9

#### Prevalence of Psychological Burden

**Figure 2:**
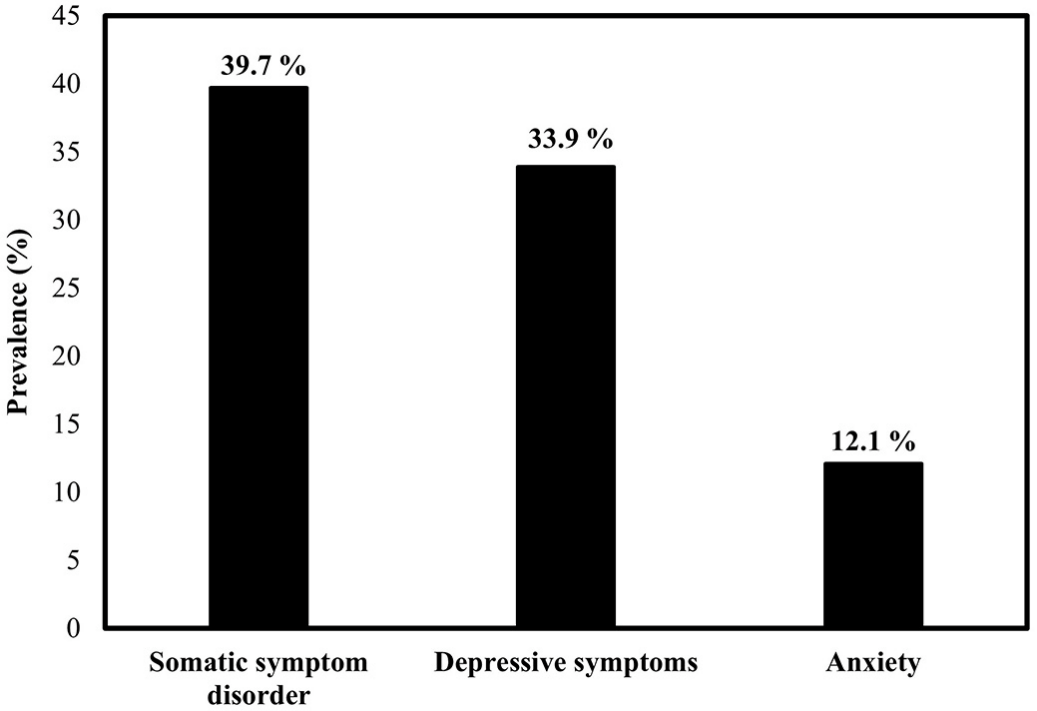
Prevalence of somatic symptom disorder, depressive symptoms, and anxiety.

Ninety-eight respondents reached the cut-off value for somatic symptom disorder (*M* = 11.3, *SD* = 9.2, and *Range* = 0–41), 76 reached the cut-off value for clinically significant depressive symptoms (*M* = 7.5, *SD* = 5.2, and *Range* = 0–21), and 27 reached the cut-off value for clinically significant anxiety (*M* = 4.4, *SD* = 4.4, and *Range* = 0–21). Thus, 121 respondents were categorized as psychological burden (= respondents with somatic symptom disorder or/and depressive symptoms or/and anxiety, Figure 2).

### Differences in Stress Experience Due to Psychological Burden

**Figure 3:**
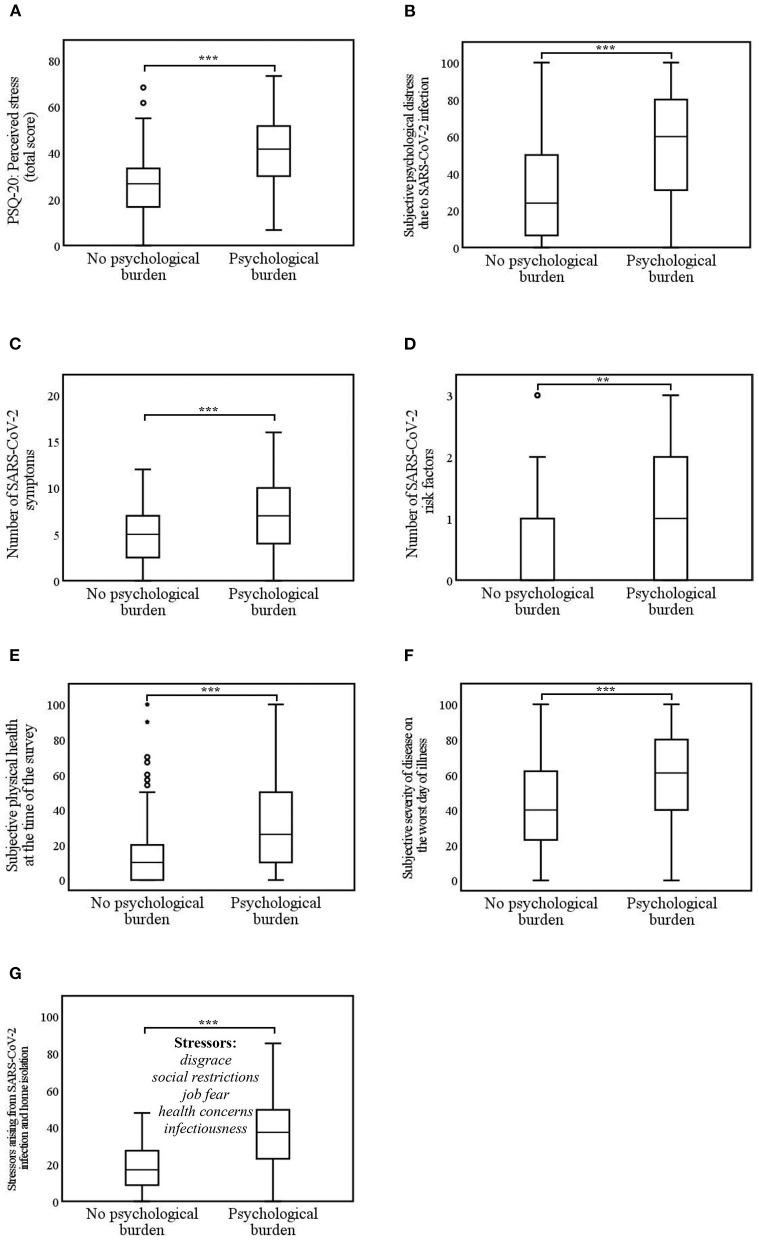
**(A–G)** Differences in stress experience due to psychological burden during SARS-CoV-2 infection and home isolation. Psychological burden = home-isolated SARS-CoV-2 infected individuals with somatic symptom disorder and/or depressive symptoms and/or anxiety. **p < 0.01; ***p < 0.001. **(A)** 0 = low perceived stress to 80 = high perceived stress; **(B)** 0 = very low stress level to 100 = very high stress level; **(E,F)** 0 = not ill at all to 100 = very severely ill; **(G)** 0 = not stressed at all to 100 = extremely stressed.

Different facets of stress experience regarding psychological burden that occurred in the context of home isolation during SARS-CoV-2 infection were investigated (Figure 3).

(1) *Perceived stress:* SARS-CoV-2 infected individuals with psychological burden (*Mdn* = 41.7) experienced significantly more perceived stress (higher PSQ-20 score) than respondents without psychological burden (*Mdn* = 26.7, *U* = 2,928.0, *z* = −6.8, *p* < 0.001, and *r* = 0.5). Respondents with psychological burden (*Mdn* = 60.0) experienced significantly higher levels of subjective psychological distress due the SARS-CoV-2 infection than the non-burdened group (*Mdn* = 24.0, *U* = 3,131.0, *z* = −6.4, *p* < 0.001, and *r* = 0.4).
(2) *Stress due to somatic factors:* Respondents with psychological burden reported significantly more SARS-CoV-2 symptoms (*Mdn* = 7.0) compared to respondents without psychological burden (*Mdn* = 5.0, *U* = 4,053.5, *z* = −4.5, *p* < 0.001, and *r* = 0.3). SARS-CoV-2 associated risk factors were significantly more frequent in the psychologically burdened group (*Mdn* = 1.0) than in the unburdened group (*Mdn* = 0.0, *U* = 5,034.0, *z* = −2.8, *p* = 0.006, and *r* = 0.2). The subjective physical health at the time of the survey was significantly worse among participants with psychological burden (*Mdn* = 26.0) compared to those without psychological burden (*Mdn* = 10.0, *U* = 3,514.0, *z* = −5.6, *p* < 0.001, and *r* = 0.4). The severity of disease on the worst day of illness was significantly worse among respondents with psychological burden (*Mdn* = 61.0) compared to those without psychological burden (*Mdn* = 40.0, *U* = 4,161.0, *z* = −4.3, *p* < 0.001, and *r* = 0.3).
(3) *Specific stressors arising from SARS-CoV-2 infection and isolation:* Respondents with psychological burden (*Mdn* = 37.3) perceived stressors arising from SARS-CoV-2 infection and isolation significantly more strongly than respondents without psychological burden (*Mdn* = 17.0, *U* = 4,362.5, *z* = −7.4, *p* < 0.001, and *r* =0.5). These differences are reflected in all stressors (disgrace, social restrictions, job fear, health concerns, and infectiousness).

### Differences in Coping Strategies Due to Psychological Burden

**Figure 4:**
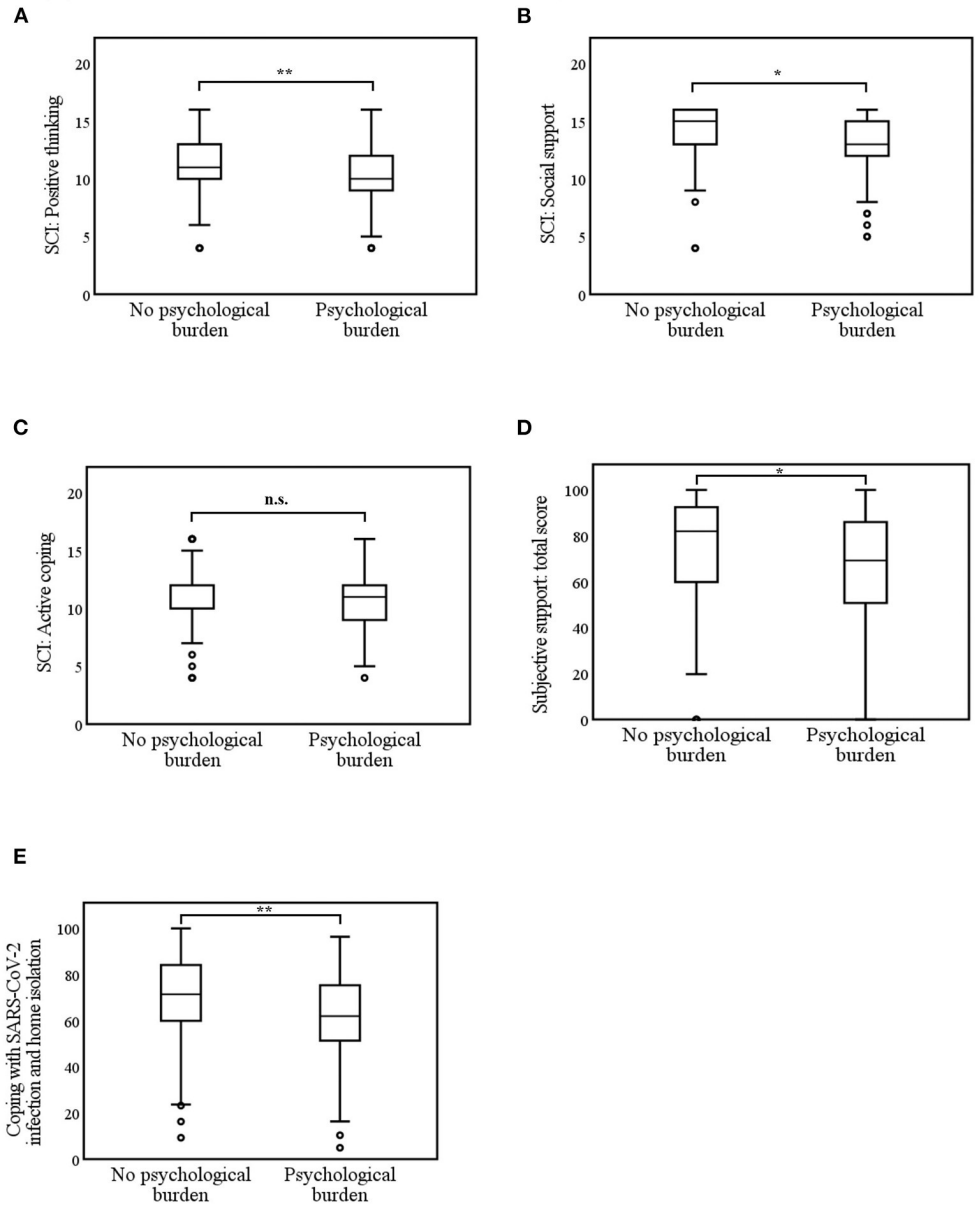
**(A–E)** Differences in coping strategies due to psychological burden during SARS-CoV-2 infection and home isolation. n.s. (not significant) *p* > 0.5; *p < 0.05; **p < 0.01; ***p < 0.001. **(A–C)** 0 = low coping skills to 20 = high coping skills. **(D)** Subjective support by social environment, employer, family doctor, health office; 0 = no support at all to 100 = full support; **(E)** 0 = low coping skills to 100 = high coping skills.

The differences in coping strategies depending on psychological burden used in the context of home isolation during SARS-CoV-2 infection were investigated next (Figure 4).

(1) *Coping strategies in general (SCI):* Respondents with psychological burden (*Mdn* = 10.0) used positive thinking significantly less as coping strategy than respondents without burden (*Mdn* = 11.0, *U* = 4,740.5, *z* = −3.1, *p* = 0.002, and *r* = 0.2). The coping strategy social support was used significantly less by the psychologically burdened participants (*Mdn* = 13.0) compared to the non-burdened participants (*Mdn* = 15.0, *U* = 5,038.5, *z* = −2.5, *p* = 0.01, and *r* = 0.2). However, active coping did not differ between the burdened (*Mdn* = 11.0) and unburdened groups (*Mdn* = 12.0, *U* = 5,316.0, *z* = −1.9, *p* = 0.06, and *r* = 0.1).
(2) *Subjective support during SARS-CoV-2 infection and isolation:* Infected respondents with psychological burden (*Mdn* = 69.3) felt significantly less supported by social environment, employer, family doctor, and health office (= support total score) than infected persons without psychological burden (*Mdn* = 82.0, *U* = 5,000.0, *z* = −2.5, *p* = 0.01, and *r* = 0.2).
(3) *Specific strategies for coping with SARS-CoV-2 infection and isolation:* Respondents with psychological burden (*Mdn* = 62.0) used significantly fewer specific coping strategies to overcome the SARS-CoV-2 infection and isolation than the unburdened (*Mdn* = 71.4, *U* = 4,362.0, *z* = −3.9, *p* < 0.001, and *r* = 0.3).

## Discussion

This is the first cross-sectional study evaluating the stress experience and the coping strategies of SARS-CoV-2 infected individuals during their home isolation and evaluating the differences regarding psychological burden. Disgrace, social restrictions, job fear, health concerns, and infectiousness were identified as stressors in the context of SARS-CoV-2 infection during home isolation. Fifty-four percent of the 224 home isolated SARS-CoV-2 infected respondents showed psychological burden. For the first time it was shown that home isolated and SARS-CoV-2 infected individuals with psychological burden perceived stressors more strongly and coped less with their infection in home isolation.

To capture these differences, screening questionnaires were initially used to screen for psychological burden. 33.9% of home-isolated SARS-CoV-2 infected individuals had a clinically significant depressiveness score, which is close to the pooled prevalence of 38% of a large meta-analysis (*n* = 4,318) on patients with SARS-CoV-2 (5). 12.1% achieved a high score for anxiety compared with 38% in the meta-analysis, in which anxiety was diagnosed at significantly lower cut-off values (5) likely explaining this difference. 39.7% of the SARS-CoV-2 infected individuals achieved a high somatic symptom disorder score, which is significantly higher than the 5.4% individuals with somatic symptom disorder of the general German population according to a cross-sectional study (*n* = 2,531) (43). Both SARS-CoV-2 infection and the prolonged pandemic could be risk factors for the development of somatic symptom disorder (44) and could explain the significantly increased prevalence. Thus, more than half (54%) of SARS-CoV-2 infected and home-isolated study participants experienced psychological burden, and overall, this is likely to persist beyond the infection and isolation phase (45, 46).

Our study shows that home isolation in the setting of SARS-CoV-2 infection is associated with the following massive stressors: disgrace, social restrictions, job fear, health concerns, and infectiousness. A qualitative-retrospective study (*n* = 64) from Finland also reported stigmatization during SARS-CoV-2 domestic isolation, along with boredom as another stressor (14). In the Finish study, boredom was likely reported by potentially non-infected quarantined contacts and did not play a role as a stressor in our study because only infected individuals were included here. During home isolation, separation of family members is often not possible. Thus, feelings of guilt regarding possible infection are pervasive at home compared with the hospital setting. Those isolated at home continue to be very concerned that the disease could take a severe course (14). The great concern about a severe course is triggered by both media reports and the lack of medical care compared to hospitalized isolation. Fear of negative consequences at work was also captured in the present study, highlighting the far-reaching effects of isolation and infection.

Among psychologically burdened individuals, the experience of stress during home isolation differed significantly and with a large effect. All stressors were perceived as more severe. This also applies to the perception of physical symptoms as stressors. Psychologically burdened individuals reported significantly more SARS-CoV-2 associated symptoms and poorer physical health. A similar relationship has already been shown in patients with a mild SARS-CoV-2 course (*n* = 895) (47). The situation of being infected with SARS-CoV-2 and isolated at home carries a threatening mix of stress that particularly affects those with psychological burden.

We also showed that SARS-CoV-2 infected individuals with psychological burden use significantly fewer coping strategies to overcome their infection and home isolation. Furthermore, they feel significantly less supported by their social environment. However, social support acts as a protective factor against psychological burden from the pandemic in non-infected individuals with preexisting depression, according to a prospective cohort study (*n* = 1,928) (48). This patient group benefits particularly from external support in the context of the SARS-CoV-2 pandemic (48). In contrast, our study identified that psychologically burdened infected respondents failed to use sufficient adaptive coping strategies, such as maintaining social contacts, adhering to a daily structure, and getting adequate sleep. Adaptive coping strategies also work as a resilience factor (49). Our study reinforces this hypothesis, as patients without psychological burden used significantly more adaptive coping strategies. The use of adaptive coping strategies as a resilience factor could explain why, in the same threatening situation, some people feel psychologically burdened and others do not.

Previous studies on coping strategies of SARS-CoV-2 infected patients have only dealt with hospitalized patients (15–17). Thus, SARS-CoV-2 infected patients (*n* = 66) showed fewer depressive symptoms when they sought the help of medical staff during mandatory hospital isolation (17). In a qualitative study (*n* = 100), it was shown that during hospitalized isolation, direct communication with other patients is an important coping strategy (16). However, these are both coping strategies that were not available in the home isolation. Psychologically burdened patients did not manage this sufficiently in the home isolation and thus bear the risk of developing even long-term psychological burden and/or (neuro)psychiatric diseases.

At the time of the study, the neuropsychiatric aspects of SARS-CoV-2 infection had not yet been discussed in the present depth. The questionnaire was based on the symptom list of the Robert Koch Institute at that time (German public health institute). In our study population, neuropsychiatric symptoms (such as headache, increased sleepiness/tiredness, disturbance in the sense of smell) were among the most common symptoms of SARS-CoV-2 infection. A meta-analysis showed that cognitive deficits, sleep disturbances, and fatigue persist beyond acute SARS-CoV-2 infection (50). Post-traumatic stress symptoms are common (45) and originate from the acute infection. Risk factors are the severity of the acute infection, but also psychosomatic aspects, such as anxiety and depression (50). The neuropsychiatric and psychiatric symptoms could in turn lead to an increased stress experience and reduced coping abilities, making it even more difficult to overcome the acute situation. Longitudinal studies are needed to further investigate this relationship.

It may be helpful to consider the SARS-CoV-2 infection in a biopsychosocial context. Biological aspects directly influence psychosocial (long-term) outcome, such as anxiety, depression, and insomnia (51). In our study, this was manifested by the SARS-CoV-2-associated risk factors. These were significantly more frequent in the psychologically burdened group. Other biological elements, such as genetics and the immune system, may also cause a corresponding vulnerability to mental illness (51). Psychoneuroimmune and psychoneuroendocrine components are suspected to negatively affect mental health acutely and as long-term consequences (52). If these associations are further explored by additional studies, they could become key elements in preventing SARS-CoV-2-related mental illness through targeted therapy.

Psychotherapy should play a central role in the treatment of psychologically burdened patients with SARS-CoV-2 as well and may help alleviate psychiatric symptoms (53, 54). The major challenge is social distancing, which is obligatory in acute SARS-CoV-2 infection. Here, online mental health services, telepsychotherapy, and telepsychiatry can overcome this situation (55). In the acute situation, they may not be as helpful and preferred as regular psychotherapy (56), so aftercare programs and psychosocial support programs are essential to cope with the consequences of the SARS-CoV-2 infection.

Psychopharmacology may be indicated to overcome the acute situation and to avoid (neuropsychiatric) long-term consequences (57). However, because SARS-CoV-2 infection is a multiorgan disease with altered pharmacokinetics, psychopharmacologic treatment is complex (58). Furthermore, the drugs used to treat SARS-CoV-2 infection, such as remdesivir, chloroquine, or interferon, are suspected to have psychotropic effects (58). Further studies are needed to responsibly use the opportunities of psychopharmacology in SARS-CoV-2 infection.

This is the only study so far focusing on stress experience and coping behaviors during home isolation and eliciting differences in terms of psychological burden. Nevertheless, there are a few limitations in the sample selection and generalization. Persons in inpatient care facilities were excluded from the study because in the pandemic setting, completion of the online questionnaire by this group would have been hardly possible. As in previous studies, validated questionnaires were used for the diagnosis of psychological burden (5, 8). However, these were self-completed by the infected individuals and therefore prone to reporting bias. Moreover, symptoms were assessed but no clinical diagnoses were made. Here, clinical interviews would have been necessary. The self-generated questionnaires on stress experience and coping behavior during home isolation have proven to be reliable, but they have not undergone a validation process.

To confirm and better generalize these study results, further, supra-regional, prospective, and follow-up studies including people in inpatient care facilities, demented patients, patients with mild cognitive impairment, and minors, are needed.

For the first time, this study provides insights into the distinct stress experience and coping strategies of SARS-CoV-2 infected individuals with a focus on home isolation. Psychologically burdened subjects experience this situation as significantly more stressful and use fewer coping strategies to manage their infection during home isolation. Accordingly, a structured support service for SARS-CoV-2 infected individuals should be established that considers the special circumstances of home isolation. Initially, all home-isolated infected individuals could benefit from low threshold coping skills interventions, such as eHealth interventions (59–62). Since the prevalence of psychological burden is 54%, a general and standardized screening procedure for psychological burden among people with SARS-CoV-2 should be established. SARS-CoV-2 infected individuals with psychological burden should receive targeted support with professional help (63) in the areas of stress experience and coping skills during their home isolation to avoid long-term mental health consequences. It is very important that psychosocial care for patients with SARS-CoV-2 is based on guidelines and protocols (64, 65). Immediate assessment of individual concerns and stressors, resource-based psychosocial support and strengthening of coping skills should be employed to prevent negative long-term effects.

## Data Availability Statement

The raw data supporting the conclusions of this article will be made available by the authors, without undue reservation.

## Ethics Statement

The studies involving human participants were reviewed and approved by Ethics Committee of the University Hospital Tuebingen. The patients/participants provided their written informed consent to participate in this study.

## Author Contributions

EK, JG, AnS, SZ, and AxS: study design. EK: data collecting and analyzing and manuscript. JG, AnS, SZ, AxS, and EK: revising manuscript. All authors contributed to the article and approved the submitted version.

## Funding

We acknowledge support by Deutsche Forschungsgemeinschaft and the Open Access Publishing Fund of the University of Tübingen.

## Conflict of Interest

The authors declare that the research was conducted in the absence of any commercial or financial relationships that could be construed as a potential conflict of interest.

## Publisher's Note

All claims expressed in this article are solely those of the authors and do not necessarily represent those of their affiliated organizations, or those of the publisher, the editors and the reviewers. Any product that may be evaluated in this article, or claim that may be made by its manufacturer, is not guaranteed or endorsed by the publisher.
